# Design and Fabrication of Smart Diapers with Antibacterial Yarn

**DOI:** 10.1155/2017/8046134

**Published:** 2017-06-22

**Authors:** Jia-Horng Lin, Bing-Chiuan Shiu, Ching-Wen Lou, Yuan-Jen Chang

**Affiliations:** ^1^Minjiang University, Fujian, China; ^2^School of Chinese Medicine, China Medical University, Taichung, Taiwan; ^3^Department of Fashion Design, Asia University, Taichung, Taiwan; ^4^Laboratory of Fiber Application and Manufacturing, Department of Fiber and Composite Materials, Feng Chia University, Taichung, Taiwan; ^5^School of Textiles, Tianjin Polytechnic University, Tianjin, China; ^6^Graduate Institute of Biotechnology and Biomedical Engineering, Central Taiwan University of Science and Technology, Taichung City, Taiwan; ^7^Department of Management Information Systems, Central Taiwan University of Science and Technology, Taichung, Taiwan

## Abstract

In this study, intelligent eco-diapers are made by combining antibacterial yarns coated with quaternary ammonium salts with conductive yarns to improve caretaking for urinary incontinence. The combination of conductive yarns and sensors can detect the moisture content in eco-diapers, and an alarm is sent when moisture is significant. A wireless module is used to send detected signals to a smartphone or tablet PC via the Internet. This concept is used for a scenario in which nurses do not randomly check on patients in a long-term care institution. When used offline, eco-diapers can send caregivers an alarm for the need to change diapers via cell phones. The diameters of the copper and silver-plated copper fibers are 0.08 and 0.10 mm, respectively. Cotton yarns are twisted with copper and silver-plated copper fibers to form the conductive yarns, which are 0.12 mm in diameter. Moreover, 30-count cotton and 150 D nylon yarns are coated with quaternary ammonium salt via dyeing and finishing processes to form antibacterial yarns. In the current study, intelligent eco-diapers are tested for their electrical and antibacterial properties as specified by AATC and JISL test standards.

## 1. Introduction

The past two decades witnessed the proposal of enormous health-care systems which benefited from emerging technologies. Telemedicine refers to proactive research that particularly combines biomedical engineering and information technology; it can provide high-quality health care without geographical constraints [1]. Urinary incontinence wastes medical human resources and social costs; it afflicts patients in a vegetative state and infants who are physiologically dependent on others. When diaper wearers are unattended for a long time, their skin may suffer from secondary damage caused by bacterial infection. Therefore, an intelligent health-care system is highly demanded by these diaper users [2].

Researchers in this field have sought out innovative improvements to address this dire situation and new high-quality techniques to create a future health-care system that is preventive, predictive, preemptive, personalized, pervasive, participatory, and patient centered [3]. With the rapid development of the Internet of things, wireless wearable sensors, which are utilized to sense vital signs in the field of medical care, became a crucial focus. These sensors can monitor the health status of patients around-the-clock to discover medical problems, such as apnea, arrhythmias, hypoxemia, and potential complications, at an early stage [4]. Smart textiles are soft and comfortable; they can also transmit the physiological signals of their users. Smart textiles allow for long-term wear and health monitoring of physiological signals, body temperature, electrocardiogram, respiration, and blood oxygen saturation degree [5, 6].

To combine electronic components and smart textiles, the textiles should have electronic conduction that can activate electronic components. Numerous studies emphasized the rendering of textiles with conductive effects. Previous studies coated conductive polymers onto the yarns to make the yarns electrically conductive. The conductive yarns are then combined with sensors to detect differences in temperature and humidity. However, these yarns failed to withstand repeated washing; their manufacturing was complex [7, 8]. Thus, the modification of fibers and yarns became another emphasized point of research. For example, polypyrrole is a piezoresistive material combined with rubbers to create elastic, electrically conductive yarns [9]. Elastic polyester and carbon-coated fibers were made into functional conductive yarns whose electrical resistance is variable under different stretching levels [10]; these studies were conducted to detect respiration. Moreover, the combination of sensors and textiles produced softness, conformability, transparency, and tensile strength [11]. Therefore, the electronic textiles formed from textiles and electronic devices included considerable potential applications.

Cloth diapers, also called eco-diapers, can bear repeated washing and are more eco-friendly than disposable diapers. However, eco-diapers easily nourish bacterial growth that damages the skin of diaper users [12]. Eco-diapers are composed of three layers. Both the top and bottom layers are made of nonwoven fabrics, whereas the interlayer consists of woven fabrics. Nonwoven layers are directly exposed to bodily fluid; therefore, they should withstand repeated washing. The interlayer should be moisture absorbent to prevent the leakage of bodily fluid [13]. In this study, the woven-fabric layer is equipped with a humidity detection function; this layer also includes an alarm that alerts the caregiver of the need to change a patient's diapers via mobile phone, web page, or auditory sound. In addition, the textiles for wearable technology should have conductive fibers to sense conductivity. Therefore, copper and silver-plated copper fibers with different diameters are used and tested for humidity resistance. The observation of their humidity resistance is important for the designs of sensors. Moreover, antibacterial cotton and nylon yarns are coated with quaternary ammonium salts to provide diapers with antibacterial effects. These two artificial and natural fibers are commonly used in daily life. The eco-diapers proposed in this study have antibacterial properties and a moisture detection function. Thus, the proposed diaper can be used in many health-care systems.

### 1.1. Design Concept

Eco-diapers for infants 24 months old and younger are commonly used in India and China followed by the Philippines and Japan. Disposable diapers for babies are preferred in other countries outside of Asia. In addition, the willingness of the vegetative, the elderly, and the physically challenged patients to use eco-diapers remain uninvestigated. The consumption of disposable diapers for infants is about 6–8 units daily; the total amount of diapers used for infants or required by hospices, hospitals, and care centers amounts to tremendous wastes daily.

In contrast, eco-diapers have the advantage of repetitive use, whereas it is disadvantaged by its need for repeated washing. The washing eco-diameter is inconvenient, which makes eco-diapers unpopular. However, it is economical and environmentally friendly. Neither eco-diapers nor disposable diapers have a mechanism for an immediate changing reminder. Using a diaper on a long-term basis causes several problems, such as skin diseases. However, adding the moisture alarm system provides users with an improved quality of life and disease prevention.

Many smart diapers are commercially available. For example, Indiegogo smart diapers have embedded a flexible print circuit and sensor system, featuring a QR code that shows diaper condition. This design is eye catching but not eco-friendly, as each diaper results in more electronic wastes. In this study, metallic fibers and other yarns are transformed into wrapped yarns, which can be efficiently made into fabrics using a loom without changing the product process. The metallic wrapped yarns are combined with a sensor that detects the moisture level of diapers. The sensor can be removed and recycled when diapers are discarded. Therefore, the proposed eco-diapers are comprised of the top and bottom nonwoven layers and a woven interlayer. The absorbent interlayer is made of two parallel metallic fibers; this layer senses humidity. When the moisture retained in the interlayer reaches saturation, the metallic fibers have a short circuit that actuates the sensors. In addition, different water contents lead to varying resistance levels; this layer can thereby signal the wetness level. Two antibacterial yarns of cotton and nylon fibers are coated with quaternary ammonium antibacterial agents through the dyeing and finishing processes. Cotton and nylon fibers are commonly used in previous research. These fibers are utilized in this study to extend the applications of antibacterial yarns to clothing and diverse livelihood commodities; these yarns decrease wound infection, skin allergy lesions, and bacteria-caused diarrhea.

### 1.2. Preparation of Samples

**Figure 1:**
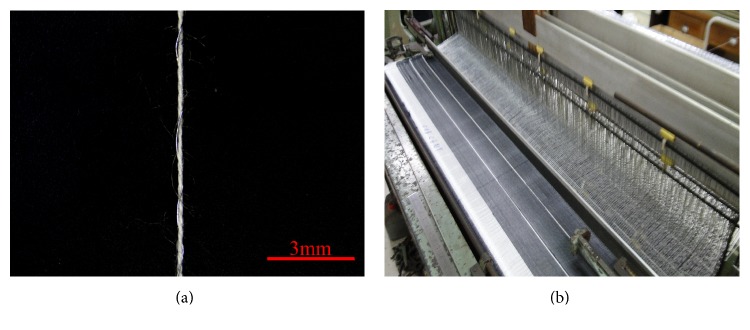
(a) Stereomicroscopic image (8x) of the twisted yarns and (b) the weaving pattern of the Rapier. The twisted yarns are composed of a 5-count cotton yarn and a metallic fiber.

A rotor twister machine is used to twist metallic and cotton fibers, as indicated in Figure 1(a); this twisting allows for the incorporation of metallic fibers with woven fabrics via a loom. The specified twist count is 4 turns/cm. A 2.6 m wide Rapier is shown in Figure 1(b); Rapier is used to prepare woven fabrics. The 1000 D high-strength PET yarns serve as warp yarns. To achieve a comparable fineness to that of the warp yarns and attain a quality structure, 500 D antibacterial nylon yarn and 30-count antibacterial cotton yarn are twisted to serve as weft yarns. The process of the proposed smart textiles in the current study is presented in Figure 1(b). Two white cotton yarns are arranged parallel to one another at 6 cm apart; these yarns are wrapped with metallic fibers to sense humidity. The black yarns are antibacterial nylon or cotton yarns. The woven fabrics are cut into 38 cm × 12 cm which is the size of commercially available Cortex eco-diapers.

### 1.3. Antibacterial Tests

The antibacterial tests include the quantitative and qualitative tests that followed AATCC-100 and JISL1902, respectively. Gram-positive *Staphylococcus aureus* and Gram-negative *colibacillus* are both used for these tests. The experimental group is the woven fabrics; the control group is the cotton woven fabrics. For the qualitative test, the inhibition zone width is used to evaluate the antibacterial effectiveness of the samples. For the quantitative tests, the bacterial reduction percentage (BR%) is computed using (1) to determine the antibacterial effectiveness of the samples.

(1) $$\begin{array}{c} BR\%=\frac{B-A}{B}\times 100\%, \end{array}$$

where *A* is the number of colonies in the experimental group and *B* is the number of colonies in the control group.

### 1.4. Electrical Property Evaluations

The metallic fibers, namely, the copper and silver-plated copper fibers, have diameters of 0.12, 0.10, and 0.08 mm. Copper has high conductivity and low cost. However, it becomes rusty and loses electrical conductivity in long-term use. Thus, copper fibers are coated by silver via electroplating to prevent oxidation and to improve conductivity. Two identical metallic fibers are arranged parallel to each other at 6 cm apart in the woven layer of the eco-diapers; the woven layer absorbs the maximum amount of water in a commercially available eco-diaper with a size of 38 cm × 12 cm. The moisture content of the woven textile is computed by (2). The woven fabric is immersed in water until saturation; its weight is TM_100%_. The weight of the dry fabric (TM_0%_) is deducted from TM_100%_ to yield the total water content. Afterwards, an Agilent M3500A (Digital Multimeter 6½ Digit, Agilent, US) is used to measure the electrical resistance caused by the short circuit between the two metallic fibers. Different electrical resistance refers to the different moisture levels of the woven fabrics; based on these results, sensors can be designed for different materials with various diameters.

(2) $$\begin{array}{c} Wt\%=\frac{{TM}_{100\%}-{TM}_{Measure}}{{TM}_{100\%}}\times 100\%. \end{array}$$

## 2. Results and Discussion

### 2.1. Structure of Diapers

The structural integrity of the cotton- and nylon-woven fabrics are observed using a stereomicroscope (SMZ-10A, Nikon Instruments Inc., Japan). In this study, the warp yarn is a 1000 D high-strength PET yarn; the weft yarn is the 30-count antibacterial cotton or 500 D antibacterial nylon yarn to prevent the oversized interstices caused by the interlacing of warp and weft yarns with uneven fineness. These interstices influence the water absorption of the woven fabrics. Two plies of identical yarns are combined to create weft yarns, which are then combined with the warp yarn to form woven fabrics. A stereomicroscope is used to observe the structure of the nonwoven fabrics; humidity resistance and antibacterial effect tests are subsequently performed on the nonwoven fabrics.

**Figure 2:**
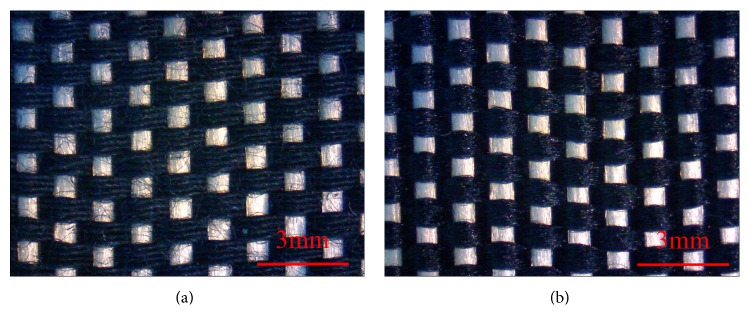
Stereomicroscopic images (8x) of (a) antibacterial cotton- and (b) nylon-woven fabrics.

Figure 2(a) presents the nonwoven fabrics made of antibacterial cotton yarns. Black cotton fibers have irregularities because they are natural fibers. They also have softness, moisture permeability, thermal insulation, and launderability.  Figure 2(b) shows the nonwoven fabrics made of antibacterial nylon yarns. The white high-strength PET yarns and black antibacterial nylon yarns both have regularity because they are man-made fibers. The nylon fibers are filaments; they are durable, stiff, and tactile and appear fine. The cotton- and the nylon-woven fabrics have comparable structures. Moreover, quaternary ammonium antibacterial agents are coated onto the cotton and nylon fibers to provide wearable smart textiles with antibacterial properties in addition to their electronic functions.

### 2.2. Antimicrobial Effectiveness

**Figure 3:**
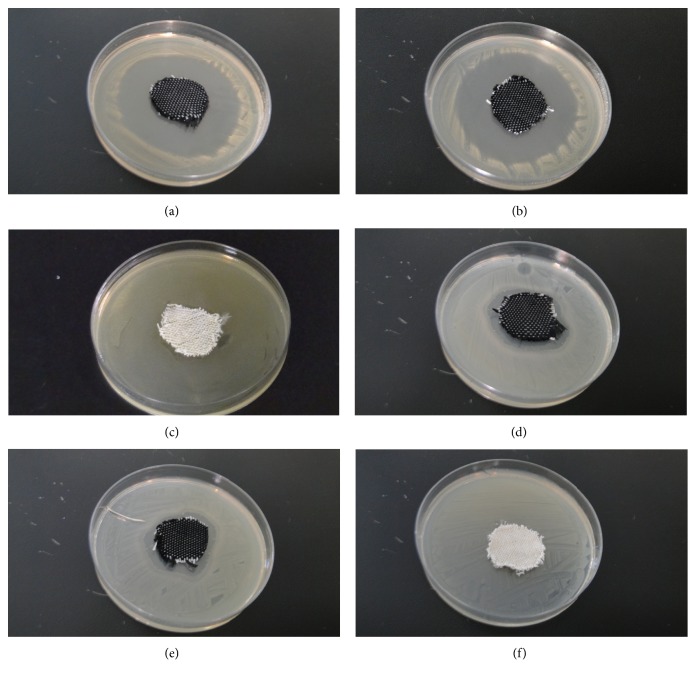
Qualitative antibacterial tests of (a) antibacterial cotton-woven fabrics, (b) antibacterial nylon-woven fabrics, and (c) cotton-woven fabrics against *Staphylococcus aureus*; qualitative antibacterial tests of (d) antibacterial cotton-woven fabrics, (e) antibacterial nylon-woven fabrics, and (f) cotton-woven fabrics against *colibacillus*.

The qualitative test results for both antibacterial cotton- and nylon-woven fabrics are presented in Figure 3. The samples and an agar medium with *Staphylococcus aureus* or *colibacillus* are placed in an incubator at 37°C for 24 h. Compared with Figures 3(a), 3(b), 3(d), and 3(e), the inhibition zone against *Staphylococcus aureus* in antibacterial cotton- or nylon-woven fabric is more significant than that against *colibacillus*. In contrast, Figures 3(c) and 3(f) indicate that common cotton-woven fabrics lack antibacterial effects against both *Staphylococcus aureus* and *colibacillus*. Quaternary ammonium salt has positive ions that easily combine with anions. Thus, bacterial membranes or their cell walls are damaged; this damage eventually causes the death of bacteria. The qualitative test is a macroscopic observation of inhibition zones. The inhibition zone of antibacterial cotton- or nylon-woven fabric ranges from 2 to 2.5 cm, whereas the inhibition zone of antibacterial cotton- or nylon-woven fabric ranges from 0.5 to 0.8 cm. Given that qualitative test results only indicate the size of inhibition zones, a digitized report corresponding to the quantitative tests is provided to indicate the bacterial reduction percentage.

**Figure 4:**
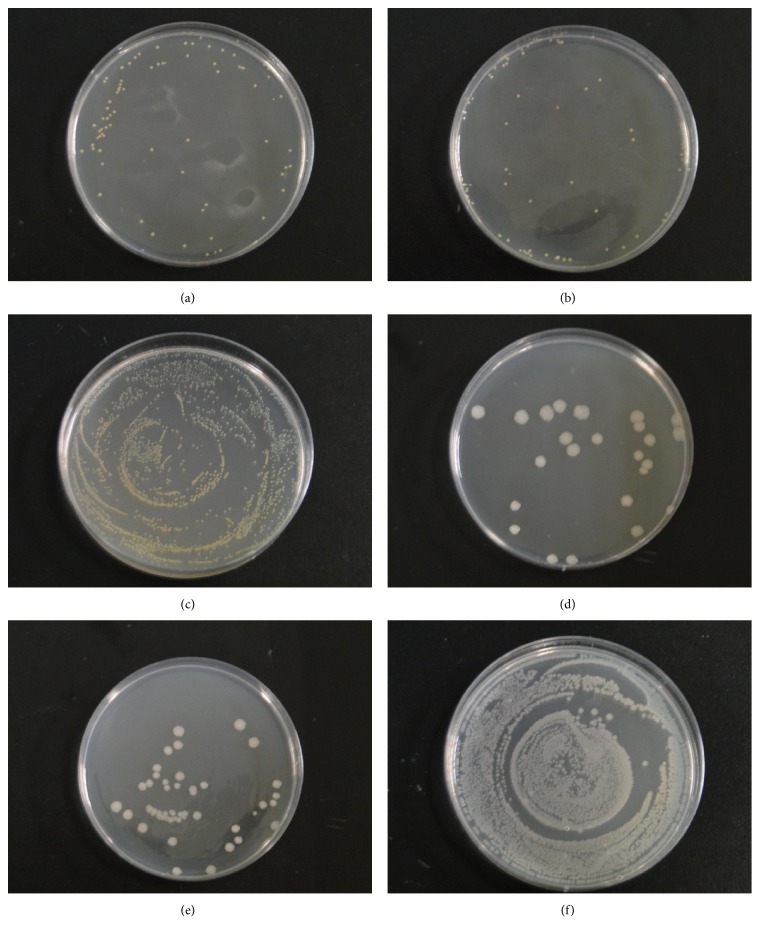
Quantitative antibacterial tests of (a) antibacterial cotton-woven fabrics, (b) antibacterial nylon-woven fabrics, and (c) cotton-woven fabrics against *Staphylococcus aureus*; quantitative antibacterial tests of (d) antibacterial cotton-woven fabrics, (e) antibacterial nylon-woven fabrics, and (f) cotton-woven fabrics against *colibacillus.*

Based on the standard operating procedures, the broth is dripped onto the samples which are then placed in Erlenmeyer flasks. The flasks are placed in an incubator at 37°C for 24 h. The bacteria are rinsed off the samples using 100 mL of sterilized deionized water. Next, 1 cc of this deionized water containing bacteria is added to an agar medium, which is then observed after 24 hours. The test results against *Staphylococcus aureus* are reported in Figures 4(a), 4(b), and 4(c). Different numbers of colonies exist between the experimental and control groups. Both cotton- and nylon-woven fabrics have a bacterial reduction percentage > 99%; this percentage is computed using (1). In addition, the quantitative test results against *colibacillus* are presented in Figures 4(d), 4(e), and 4(f). Similarly, both cotton- and nylon-woven fabrics have a bacterial reduction percentage > 99% against *colibacillus*. Quaternary ammonium salt-type yarns exhibit excellent antibacterial effect; they are placed between two nonwoven fabrics combined with the moisture-absorbent layer in this study. They are not in direct contact with the skin; thus, they do not negatively influence the human body in terms of convulsions, hypotension, nausea, and vomiting.

### 2.3. Electrical Resistance

Table 1 indicates the humidity sensitive resistance of the cotton- and nylon-woven fabrics containing copper or silver-plated copper fibers. The metallic fibers have three diameters, namely, 0.08, 0.1, and 0.12 mm. Copper fibers are commonly used for conductivity in various circuits. They have the disadvantage of easy oxidation that changes their resistance. Therefore, silver-plated copper fibers are used for higher stabilities compared with copper fibers. When containing the same diameter of metallic fibers, cotton-woven fabrics have lower electrical resistance than nylon-woven fabrics; this difference is ascribed to the high-moisture absorption of cotton fibers. However, natural fibers experience fatigue after repeated washing, which in turn decreases their moisture absorption. Compared with natural fibers, synthetic fibers have high stability, launderability, and chemical resistance. Metallic fibers are subject to breakage due to the diameter in millimeter, which requires complex adjustments for the fabrication. In this study, different combinations of the two antibacterial woven fabrics and two metallic fibers are evaluated in terms of their moisture contents and their corresponding electrical resistance. Thus, sensors can be designed according to the electrical resistance caused by different woven fabrics to notify users of the hygroscopic level of eco-diapers.

**Figure 5:**
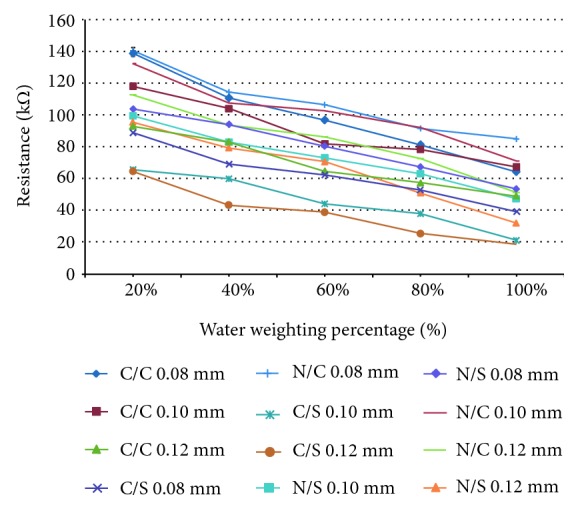
Resistance of nylon- and cotton-woven fabrics relative to the water retention rate (%) and the diameter of silver-plated copper wire (mm).

The profiles of data listed in Table 1 can be plotted in Figure 5. Figure 5 shows the resistance of cotton- and nylon-woven fabrics relative to different water weighting percentages. Only four profiles are plotted for demonstration, that is, cotton with silver-plated copper fibers, 0.08 and 0.12 mm in diameter, as well as nylon with silver-plated copper fibers, 0.08 and 0.12 mm in diameter. A well-designed sensor means that it has a large sensitivity. In the current study, we adopted the regression line slope, as shown in (3), to calculate fabric sensitivity *S*. The *S*value can be used to evaluate the fabric performance in detecting water weighting percentage.

(3) $$\begin{array}{c} S=\frac{\sum \left(X-\bar{X}\right)\left(Y-\bar{Y}\right)}{{\sum \left(X-\bar{X}\right)}^{2}}, \end{array}$$

where *X* is the resistance and *Y* is the water weighting percentage.

The *S* values of each profile are depicted in Figure 5. Cotton fabric has better water retention property than nylon. However, the *S* value decreases when cotton is combined with large diameter metallic wires. Smaller *S* value indicates that cotton-woven fabrics are less sensitive in detecting resistance. In contrast, nylon-woven fabric has worse water retention property than cotton. However, the *S* value increases when nylon is combined with large diameter metallic wires. A larger *S* value indicates that nylon-woven fabrics are more sensitive in detecting resistance. All the *S* values of each fabric are listed in Table 1. Consequently, a higher *S* value indicates that the fabric has higher sensitivity to detect resistance.

### 2.4. Design of Sensors

**Figure 6:**
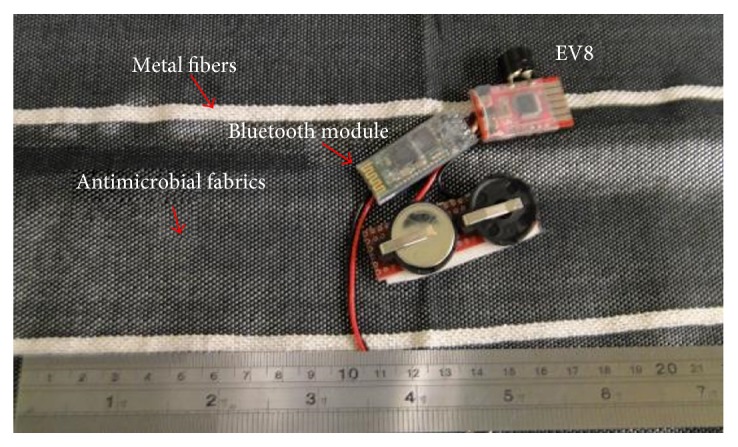
Image of the sensor and antimicrobial fabric.

As shown in Figure 6, EV8 microcontrollers (Innovati Inc., Taiwan, R.O.C.) are developed circuit boards with a smart module containing microcontrollers that execute functions and related applications. A buzzer is included in this design; an alarm goes off when the data signals detected via Bluetooth are sent from a mobile phone or a receiver to the cloud. This developed modulus uses the BASIC programming language with its main control IC supporting the repeated burns of the programming language. The electrical resistances caused by different woven fabrics are added in their corresponding program. Once the electrical resistances with combined woven fabric and metallic fibers are measured, the programs can be designed for control relative to electrical resistance based on water absorption. The data can either be sent via a Bluetooth modulus to a mobile phone or set off an alarm.

**Figure 7:**
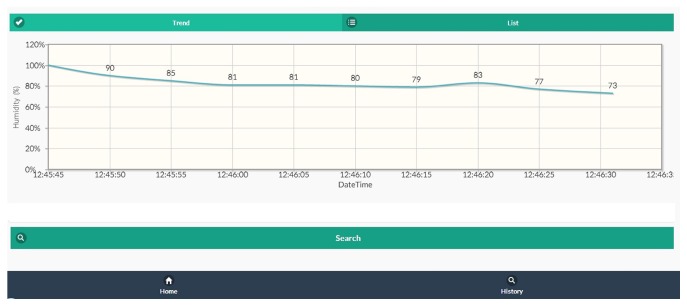
Web page indicating the humidity of smart diapers.

Taking advantage of the public cloud services, the wireless module that transmits signals to cloud servers, such as Azure, Amazon EC2, or Google Cloud, has become an indispensable tool in health care.  Figure 7 presents the web-based notification function when diaper humidity reaches 60%. The nursing station in care facilities or hospitals can become aware of the moisture level of the diapers of patients; the caregivers for infants can also receive the sensor's alarm or an alert message via mobile phones [14]. This study aims to develop eco-diapers that can be used daily in response to the needs of differently abled diaper users. This eco-diaper system requires only the smart diaper developed in the current study combined with a mobile phone without many modifications. This smart diaper incorporates metallic fibers and sensors to produce specific benefits, namely, the enhancement of the users' comfort and improvement of the users' well-being.

## 3. Conclusion

This study proposes the use of antibacterial cotton and nylon yarns to provide a novel variety of eco-diapers; these yarns are treated with quaternary ammonium salts to make them antibacterial. The antibacterial effectiveness of the woven fabrics was evaluated. The test results indicate that using quaternary ammonium salts provides a bacterial reduction percentage higher than 99% against *Staphylococcus aureus* or *colibacillus*. In addition, wearable technology became an important research direction. Previous studies have used complicated methods to form conductive textiles; this study improves these methods by combining woven fabrics and electronic products for eco-diapers. The synthesis of metallic and functional yarns highly decreases the discomfort of eco-diaper users. The nonwoven interlayer of the eco-diapers is combined with metallic yarns to sense humidity. These two parallel metallic yarns can detect different hygroscopic levels of eco-diapers and examine humidity according to electrical resistance. Regardless of whether users are the infants or the disable, comfort, outlook, and position of the tangible sensor should all be considered. In addition, a wireless transmission module is used to send an alert message to the smart phones of caregivers via a web-based notification function. Therefore, a smart diaper combined with a smart phone removes the need for caregivers to randomly check the diaper status of patients. This system can subsequently prevent skin disease. Moreover, the manpower shortage in long-term care institutions can be alleviated.

## Figures and Tables

**Table 1:** Electrical resistance of fabrics in relation to moisture content (*N* = 5).

Warp yarn/weft yarn	Stainless steel fiber types	Diameter of the stainless steel fibers	Water weighting percentage					*S* value
			20%	40%	60%	80%	100%	
			(kΩ)	(kΩ)	(kΩ)	(kΩ)	(kΩ)	
PET/cotton	Copper	0.08 mm	138.67 ± 5.51	110.75 ± 4.34	96.91 ± 0.52	81.45 ± 3.48	64.36 ± 2.18	−1.10
PET/cotton	Copper	0.10 mm	118.09 ± 6.59	104.28 ± 1.67	81.82 ± 2.65	78.53 ± 1.15	67.45 ± 1.42	−1.495
PET/cotton	Copper	0.12 mm	93.15 ± 7.97	83.06 ± 1.04	64.88 ± 7.78	57.6 ± 3.93	49.35 ± 2.56	−1.723
PET/cotton	Silver-copper	0.08 mm	88.85 ± 0.52	69.27 ± 3.12	62.38 ± 1.35	52.98 ± 1.49	39.27 ± 0.96	−1.685
PET/cotton	Silver-copper	0.10 mm	65.72 ± 1.66	59.91 ± 1.75	44.25 ± 1.25	38.01 ± 2.25	21.28 ± 0.46	−1.758
PET/cotton	Silver-copper	0.12 mm	64.69 ± 2.55	43.51 ± 1.42	38.99 ± 1.67	25.66 ± 0.62	19.32 ± 1.53	−1.766
PET/nylon	Copper	0.08 mm	140.52 ± 1.09	114.5 ± 1.31	106.52 ± 1.37	91.6 ± 1.58	85.02 ± 1.6	−1.411
PET/nylon	Copper	0.10 mm	132.33 ± 1.19	107.62 ± 0.68	102.57 ± 1.19	92.34 ± 2.17	71.25 ± 0.53	−1.383
PET/nylon	Copper	0.12 mm	112.66 ± 1.83	93.56 ± 1.50	86.27 ± 0.87	72.58 ± 1.57	51.47 ± 1.56	−1.363
PET/nylon	Silver-copper	0.08 mm	103.69 ± 1.01	94.21 ± 1.29	80.66 ± 1.23	67.52 ± 0.48	53.67 ± 1.04	−1.572
PET/nylon	Silver-copper	0.10 mm	99.53 ± 1.548	82.92 ± 1.26	72.94 ± 0.72	63.22 ± 1.28	47.58 ± 1.44	−1.537
PET/nylon	Silver-copper	0.12 mm	95.61 ± 1.01	79.65 ± 1.88	70.84 ± 0.73	51.22 ± 1.85	32.42 ± 1.84	−1.273
